# Metabolic Profiling of a Mediterranean-Inspired (Poly)phenol-Rich Mixture in the Brain: Perfusion Effect and *In Vitro* Blood–Brain Barrier Transport Validation

**DOI:** 10.1021/acs.jafc.5c02288

**Published:** 2025-04-24

**Authors:** María Ángeles Ávila-Gálvez, Beatriz Garay-Mayol, Alicia Marín, María Alexandra Brito, Juan Antonio Giménez-Bastida, Juan Carlos Espín, Antonio González-Sarrías

**Affiliations:** 1 Laboratory of Food & Health, Research Group on Quality, Safety and Bioactivity of Plant Foods, CEBAS-CSIC, Campus de Espinardo, Murcia 30100, Spain; 2 Research Institute for Medicines (iMed.ULisboa), Faculty of Pharmacy, Universidade de Lisboa, Av. Prof. Gama Pinto, Lisbon 1649-003, Portugal; 3 Department of Pharmaceutical Sciences and Medicines, Faculty of Pharmacy, Universidade de Lisboa, Av. Prof. Gama Pinto, Lisbon 1649-003, Portugal

**Keywords:** phenolic compounds, blood−brain barrier, oral gavage, microbial metabolites, metabolism, pharmacokinetic

## Abstract

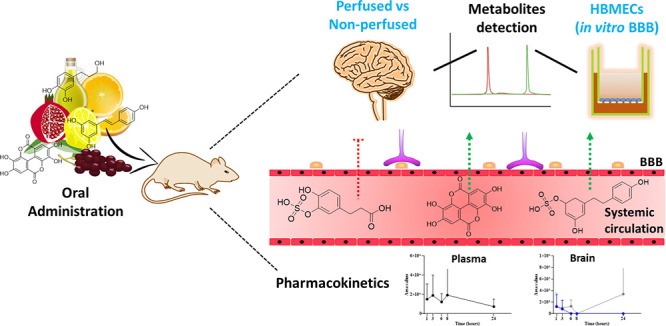

A Mediterranean diet rich in (poly)phenols has been linked to neuroprotection, but its effects likely depend on the ability of phenolic metabolites to cross the blood–brain barrier (BBB). This study evaluated the kinetics plasma and brain distribution of phenolic metabolites in Sprague–Dawley rats following oral administration of a polyphenol-rich extract mixture from Mediterranean foods (pomegranate, lemon, orange, grape, and olive). UPLC-ESI-QTOF analyses revealed 39 phenolic-derived metabolites in plasma, of which 20 were in nonperfused (NPB) and 19 in perfused brains (PB), including hydroxytyrosol and tyrosol sulfates, ellagic acid, dihydrocaffeic acid, and derived metabolites. Kinetic data showed substantially higher plasma metabolite concentrations than the brain, with slightly higher levels in NPB. The BBB transport efficiency of phenolic metabolites was validated *in vitro* using human brain microvascular endothelial cells (HBMECs), showing improved transport when tested as mixtures. These findings confirm that circulating phenolic metabolites from Mediterranean foods can reach brain tissues, contributing to preventing neurodegenerative diseases.

## Introduction

Neurodegenerative diseases (ND) such as Alzheimer’s or Parkinson’s, among others, constitute one of the most important public health problems in developed countries and are increasing as life expectancy increases. Thus, according to the United Nations, hitherto, more than 55 million people are living with dementia or Alzheimer’s worldwide and more than 10 million are diagnosed with Parkinson’s. Projections estimate that by 2050, the number of affected individuals will surpass 114 million.^1−3^ These diseases are characterized by the progressive degeneration and/or death of neurons, which leads to impaired movement or cognitive functions (dementia, thinking and behavior detriment, among others).^4,5^ Therefore, the rising incidence and prevalence of ND, together with the lack of new and effective pharmacological treatments, highlights the need for a more comprehensive understanding of how different aspects of lifestyle, such as exercise and diet, can influence brain health, supporting long-term neuronal function and cognitive performance.^6,7^ Indeed, current knowledge indicates that brain changes associated with the development of ND can begin up to two decades before the onset of symptoms, making it challenging to reverse the pathology at the time of diagnosis.^8,9^ Hence, since preventing chronic diseases is a better strategy than their treatment, reducing their risk through a healthy diet emerges as a key priority objective for health professionals, scientists and food industries.

In this regard, the Mediterranean diet, characterized by the consumption of fruits, vegetables, legumes, and whole grains, arises, according to epidemiological and observational studies, as a protective factor against many chronic diseases, including ND or cognitive decline.^7,10,11^ Notably, among the molecules present in these foods, dietary phenolic compounds or (poly)phenols are recognized as relevant players in these beneficial effects. Over the past decades, these molecules have been in the spotlight as a promising nonpharmacological approach for preventing and/or treating ND and/or cognitive decline,^12−16^ a link that is supported by recent preclinical studies. Key examples include flavonoids from citrus, stilbenes such as resveratrol from grapes and red wine, ellagic acid and ellagitannins from pomegranates, and hydroxytyrosol from olive oil.^17−23^ However, the vast majority of these studies overlook key factors such as bioavailability, metabolism and distribution in brain tissue.^24^ Unfortunately, this inaccurate approach persists even in recent studies.

Dietary (poly)phenols are poorly bioavailable and barely reach systemic tissues as they occur in food (glycosides, polymers or esters). Some (poly)phenols are absorbed in the small intestine, conjugated by phase II enzymes, and enter the bloodstream. However, most ingested (poly)phenols reach the colon intact, where they are metabolized by the gut microbiota, producing microbial metabolites, which are absorbed and extensively metabolized by phase II enzymes to enter the bloodstream. These conjugates, mostly microbial-derived metabolites, can reach systemic tissues like the brain, where they may exert a neuroprotective effect.^23,25−27^ Nonetheless, although evidence consistently shows that certain (poly)phenols improve brain function in animals,^22,28,29^ it remains necessary to determine whether these circulating phenolic metabolites can cross the blood-brain barrier (BBB). This fact would clarify whether they directly influence brain tissue or act indirectly as drivers of the observed effects.^30^

Several animal studies have identified some phenolics in the brain, such as gallic acid, ellagic acid, resveratrol and anthocyanins, as well as their phase II conjugates and (or) derived microbial metabolites, depending on the phenolic. Therefore, these compounds may be responsible for the neuroprotective effects associated with consuming (poly)phenol-rich foods. Nevertheless, these findings often stem from studies involving nondietary doses or direct intravenous administration of free (unconjugated) metabolites or nanoformulations.^31−39^ Additionally, many studies fail to perfuse brain tissues before analysis, risking contamination by blood metabolites. Brain perfusion is mandatory to confirm whether the circulating metabolites effectively cross the BBB and reach the brain cells. In studies investigating the brain distribution of dietary (poly)phenols, accurately distinguishing compounds that have traversed the BBB from those merely present in cerebral vasculature is crucial. Transcardial perfusion with isotonic saline solutions effectively removes residual blood, ensuring that detected (poly)phenols or derived metabolites are localized within brain parenchyma rather than confined to blood vessels.^40^ This technique is essential for precise assessment of compound brain distribution and for understanding their potential neuroprotective effects. Implementing perfusion protocols minimizes confounding variables, thereby enhancing the reliability of data regarding polyphenol localization and activity within the central nervous system. Another limitation is that metabolite quantification often occurs after enzymatic hydrolysis (using glucuronidase/sulfatase), making it difficult to determine their molecular forms and exact concentrations in the brain. In this line, even though the uptake of phenolic metabolites described in perfused brains from rodent models, the identification and quantification of these metabolites was carried out after enzymatic hydrolysis and therefore, the levels of these phenolic could be overestimated^41−44^ or underestimated due to incomplete hydrolysis.^45^

In this study, we conducted, for the first time, a pharmacokinetic and brain distribution analysis in Sprague–Dawley rats after oral administration of a (poly)phenol-rich extract mixture derived from Mediterranean diet foods (pomegranate, lemon, orange, grape, and olive) using human dietary doses. We assessed the *in vivo* ability of phenolic compound-derived metabolites to cross the BBB and compare them with circulating plasma metabolites. Quali- and quantitative differences between perfused and nonperfused brains were also evaluated. Finally, to corroborate the *in vivo* findings, we used an *in vitro* BBB model based on human brain microvascular endothelial cells (HBMECs). This study provides novel information on the concentrations and metabolic profile of phenolic metabolites that can reach brain tissue. Our results may pave the way for more physiologically relevant preclinical studies to determine whether these circulating metabolites contribute to the protective effect attributed to the Mediterranean diet against ND.

## Materials and Methods

### Reagents

Hesperetin 7-*O*-glucuronide and hesperetin 3′-*O*-glucuronide were obtained from Villapharma Research S.L. (Parque Tecnológico de Fuente Alamo, Murcia, Spain). Dihydroresveratrol 3-*O* glucuronide, *t*-resveratrol 3-O-glucuronide, *t*-resveratrol 3-*O*-sulfate, and *t*-resveratrol 4′-*O* sulfate were obtained as described elsewhere.^34^ The metabolite 5-(3′,4′-dihydroxyphenyl)-γ-valerolactone 3′-sulfate was kindly provided by Dr. del Rio (Univ. Parma, Italy). Dihydrocaffeic acid and caffeic acid 3-*O*-sulfate were provided by Dr. Nunes Dos Santos (NOVA Medical School, Portugal). 2,4-Dihydroxybenzoic acid, and ellagic acid were obtained from Sigma-Aldrich (St. Louis, MO, USA). Hydroxytyrosol 4’-sulfate, hydroxytyrosol 3-sulfate, tyrosol 4’-sulfate, *p*-coumaric acid 4-*O*-sulfate, 5-(3-hydroxyphenyl)-valeric acid and dihydrocaffeic acid 3-*O*-sulfate were purchased from Toronto Research Chemicals (Toronto, Canada). Ferulic acid 4-*O*-sulfate was provided by Biosynth (Compton, Berkshire, UK). 3-(3-Hydroxyphenyl) propionic acid and 3-(2-hydroxyphenyl) propionic acid were obtained from Fluorochem (Hadfield, Derbyshire, UK). All extraction and liquid chromatography solvents were UPLC-certified and were obtained from J.T. Baker (Phillipsburg, NJ, USA).

### Animals and Experimental Design

**Figure 1:**
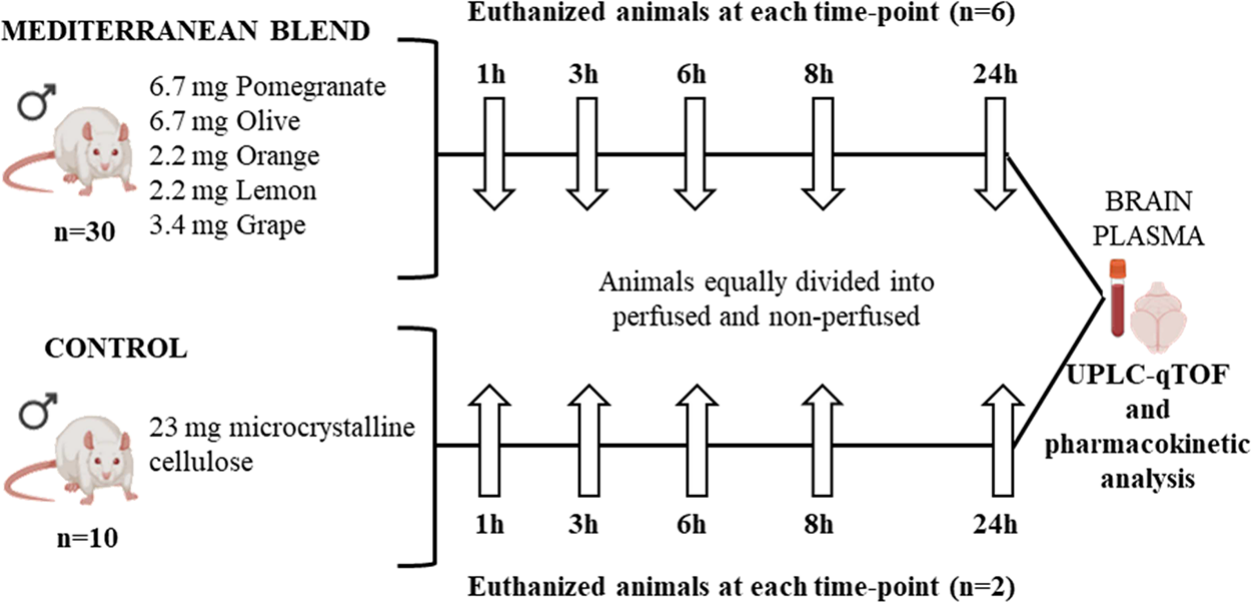
Experimental design and animal grouping. Amount (mg) of each plant extract (lemon, orange, pomegranate, grape, and olive) included in the Mediterranean blend administered to the animals is detailed.

Adult male Sprague–Dawley rats weighing 250–300 g were purchased from the Animal Experimentation Service of the University of Murcia (Murcia, Spain). All of the experimental procedures followed the Directive of the European Council 2010/63/UE and the Spanish government (RD 53/2013) and were approved by the animal ethics committee (University of Murcia, Spain) and the local government (reference 832/2022). Animals were maintained in a temperature-controlled environment with a 12 h light-dark cycle. Animals had free access to standard laboratory food and tap water. Based on the experimental design shown in Figure 1, one capsule was administered by gavage to each fasting animal. A total of 40 animals were used in the study. Thirty animals received a capsule containing a mixture of different Mediterranean food extracts, while ten animals received a capsule with microcrystalline cellulose and served as the control group. At each time point (1, 3, 6, 8, and 24 h postadministration), six animals from the extract group were sacrificed, three with transcardial perfusion using saline (PB) and three without perfusion (NPB), to assess the distribution of extract-derived metabolites in plasma and brain. To minimize animal use in accordance with the 3Rs principle, two control animals were sacrificed at each time point (one PB and one NPB). These controls served to identify potential endogenous compounds or background signals not related to the extract administration. All animals were fasted overnight prior to gavage and euthanized with carbon dioxide.

### Capsule Preparation and HPLC Analysis of Phenolic Compounds

A mixture of different Mediterranean food extracts, including lemon, orange, pomegranate, grape and olive, were kindly provided by Laboratorios Admira S.L. (Alcantarilla, Murcia, Spain). All components were encapsulated in hard gelatin capsules (size 9), specially designed to administer to rats (Torpac, Fairfield, USA). The human equivalent dose (HED)^46^ of phenolics administered to the animals was 345 mg in a 70 kg person. The content of phenolic compounds per capsule was analyzed by high-performance liquid chromatography-electrospray tandem mass spectrometry (HPLC-ESI-MS/MS) based on the methods described elsewhere.^47^ The detailed phenolic composition of the capsules (22 mg of a blend of plant-derived food extracts rich in (poly)phenols), presented in Supporting Information, Table S1, includes 18 phenolic compounds, providing an average of 8.35 ± 0.50 mg per capsule.

### Sample Collection and Phenolic Extraction

Blood samples collected by cardiac puncture in sodium heparin tubes at each time point after sacrifice were immediately centrifuged at 14,000 × *g* for 15 min at 4 °C, and the plasma was stored at −80 °C until their analysis. Plasma samples were extracted with ACN:formic acid (98:2, v/v), centrifuged and evaporated in a speed vacuum concentrator (Savant SPD14 0DDA). The evaporated samples were resuspended in 100 μL MeOH and filtered through 0.22 μm regenerated cellulose filters (Minisart Sartorius, Madrid, Spain). Next, half of the animals at each time point were perfused with ice-cold phosphate-buffered saline, and the blood-free brain was dissected out immediately and stored at – 80 °C until analysis, as previously reported.^37,48^

Approximately 400 mg of brain tissue (perfused or nonperfused) was weighed and extensively washed with cold PBS to avoid external blood contamination. After weighting, the samples were placed in 2 mL screw cap tubes. One ball of 3.2 mm and one spoon of 0.9–2.1 mm stainless steel beads (Next Advance, Averill Park, NY) were added to each tube containing weighted samples and 1.2 mL MeOH: hydrochloric acid (99.9:0.1, v/v). Next, the tubes were placed in a bullet blender homogenizer for 5 min, centrifuged at 14,000 × *g* for 10 min at 4 °C, and the collected supernatant was reduced to dryness in a speed vacuum concentrator. The evaporated samples were resuspended in 100 μL MeOH and filtered through 0.22 μm regenerated cellulose filters.

### Targeted Metabolomics Analysis by UPLC-ESI-QTOF

We performed a targeted metabolomic analysis using an ultrahigh-performance liquid chromatographic system (UPLC) coupled to a quadrupole-time-of-flight (QTOF) mass spectrometer. The phenolic compounds were separated using an Agilent Poroshell 120 column (100 mm × 3 mm, 2.7 μm) and a 5 μL injection volume. The mobile phases used were 0.1% formic acid in water (A) and 0.1% formic acid in ACN (B), and the gradient was: 0–3 min, 5–15% B; 3–11 min, 15–30% B; 11–14 min, 30–50% B, 14–16 min, 50–90% B. Finally, the B content was decreased to the initial conditions (5%) in 1 min, and the column re-equilibrated for 4 min. The flow rate was set constant at 0.4 mL/min. The spectra were acquired in the *m*/*z* range from 100 to 1100, in negative polarity mode and an acquisition rate of 1.5 spectra per second. Quantitative data were processed using the Mass HunterQualitative Analysis software (version B.08.00, Agilent). A target screening strategy was applied for the qualitative analysis of possible metabolites derived from the consumption of the Mediterranean blend. More than 150 possible compounds were browsed in the different samples (Supporting Information, Table S2). These compounds encompass derived metabolites conjugated (glucuronides, sulfates, and sulfoglucuronides, among others). The exact mass of the proposed compound was extracted using an extraction window of 0.01 *m*/*z*. Metabolite quantification was performed by integrating the peak areas of their extracted ion chromatograms (EICs). Compounds with available standards were quantified using calibration curves derived from the respective standards, while other metabolites were semiquantified based on their peak area values.

### *In Vitro* Blood–Brain Barrier Transport

The BBB transport model was established using human brain microvascular endothelial cells (HBMECs), following a methodology previously described.^49^ Cells were maintained at 37 °C in a humidified environment with 5% CO_2_. HBMECs were seeded on 12-well Transwell inserts (12 mm, 0.4 μm pore polyester membrane; Corning, Madrid, Spain) and cultured for 6 days to form confluent monolayers suitable for transport studies. Transport assays were carried out in Hanks’ Balanced Salt Solution (HBSS) supplemented with 0.1% fetal bovine serum (FBS, v/v). The cells were exposed on the apical side to individual phenolic metabolites detected in the brain from the animal study, a representative mixture containing equal concentrations of these metabolites, or a physiological mixture reproducing their relative percentages as observed in the brain. All treatments were applied at a final concentration of 2.5 μM for 2 h (Supporting Information, Table S3). The integrity of the cell monolayers was confirmed by measuring transendothelial electrical resistance (TEER) at the start of the experiment (0 h) and after the last time point (2 h) using an EVOM2 Epithelial Volt Ohm Meter (World Precision Instruments, Inc., USA).

Samples from the apical and basolateral compartments were stored at −80 °C for further analysis. Transport of the compounds was evaluated using UPLC-qTOF as described elsewhere,^38^ and transendothelial permeability expressed as a percentage of the ratio between the concentration in the basolateral compartment relative to the total concentration in both compartments.

### Pharmacokinetic Parameters and Statistical Analysis

The estimated pharmacokinetic parameters were examined by noncompartmental analysis using the PKSolver, a complement add-in software of Microsoft Excel.^50^ The following pharmacokinetic parameters were calculated: maximum peak concentration (*C*_max_), time to reach Cmax (*T*_max_), mean residence time (MRT), the total area under the curve (AUC) from the initial time point (0 h) to the final time point (24 h) and half-life (*T*_1/2_). Statistical analyses were applied depending on the experimental context. Comparisons between perfused (PB) and nonperfused (NPB) brain samples were performed using either an unpaired *t* test or the Wilcoxon signed-rank test, based on data distribution (normality assessed via Shapiro-Wilk test).

Finally, one-way ANOVA with Tukey’s multiple comparisons test was exclusively applied to in vitro BBB transport experiments. The three tested conditions for each compound (individual compound, a mixture containing equal concentrations of these compounds, or a physiological mixture reproducing their relative percentages as observed in the brain were evaluated. Plots were performed using GraphPad 9.0 (GraphPad Software, Boston, Massachusetts, USA). Statistical significance was set at *P* < 0.05.

## Results and Discussion

### (Poly)phenols and Derived Metabolites in Plasma and Brain

**Figure 2:**
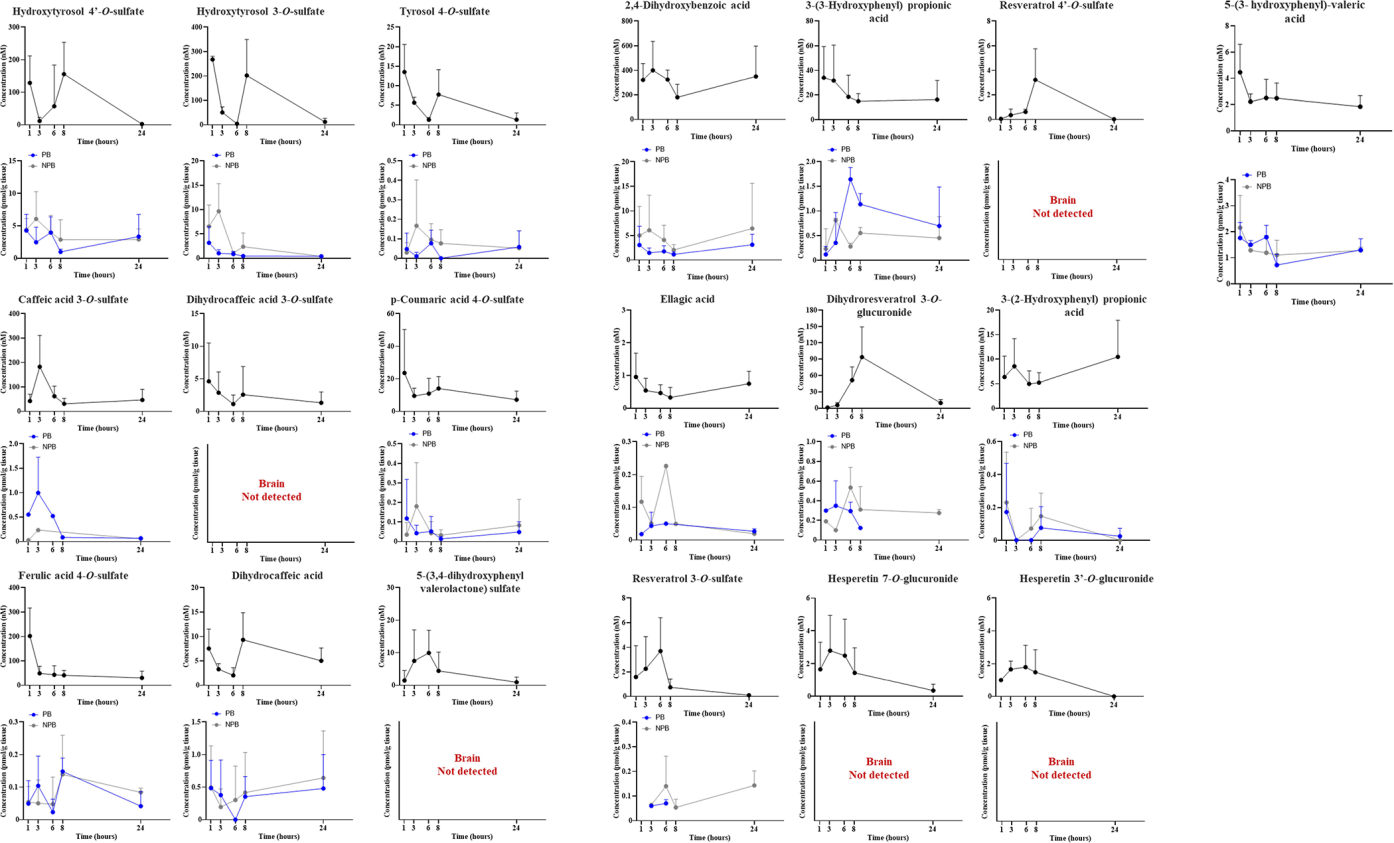
Phenolic metabolites concentration–time profiles using authentic standards in plasma (black), perfused brain (PB, blue), and nonperfused brain (NPB, gray) derived from consuming a (poly)phenol Mediterranean diet blend. Values are expressed as concentrations (nM for plasma and pmol/g tissue for brain) with standard deviations (SD). Data correspond to plasma samples (*n* = 6) and brain samples (*n* = 3) for each group of animals.

A total of 39 compounds identified in plasma showed a broad diversity of (poly)phenol-derived metabolites at the systemic level. Twenty metabolites were also detected in the brain, highlighting their potential to cross the BBB. However, of the 20 phenolic compounds present in the brain, dihydroresveratrol sulfate was only detected in the nonperfused brain (NPB) (Table 1; Figure 2). Of all the compounds identified in animals, 19 were quantified using authentic standards, and their pharmacokinetic profiles are presented in Figure 2. The concentration–time profiles of phenolic metabolites tentatively identified based on their extracted ion chromatogram (EIC) peak areas were generated for those metabolites detected in both plasma and PB, allowing a direct comparison between these matrices. These profiles, shown in Supporting Information, Figure S1, reveal that metabolites exhibited substantially higher area values in plasma compared to the brain, suggesting a selective permeability and retention mechanism at the BBB.^51^ Within the brain, the *C*_max_ values of most metabolites were slightly higher in NPB, except for homovanillic alcohol sulfate and 3-(3′-hydroxy-4′-methoxyphenyl)propionic acid, which reached higher concentrations in PB. Unfortunately, a direct comparison between metabolites is unfeasible due to the potentially significant differences in the ionization response across the molecules. In general, metabolites in NPB showed higher concentrations than PB, likely due to residual blood contamination in NPB samples. This suggests that some metabolites may be localized in brain vasculature or bound to brain endothelial cells rather than exclusively located in the brain parenchyma. Therefore, this highlights the importance of using perfusion techniques to accurately assess metabolite distribution within brain tissue, which should be considered mandatory in metabolomic studies.^40,52^

**Table 1:** (Poly)phenols and Derived Metabolites Identified in Perfused (PB) and Nonperfused Brains (NPB) and Plasma (P)

**no.**	**compounds**	**RT (min)**	*m*/*z*^*-*^**experimental**	**molecular formula**	**error (ppm)**	**score**	**occurrence**
1	tyrosol glucuronide	2.97	313.0929	C_14_H_18_O_8_	–0.53	98.19	P
2	3-(3′,4′-dihydroxyphenyl)propionic acid glucuronide	3.07	357.0827	C_15_H_18_O_10_	0.24	99.50	P
3	hydroxytyrosol 4′-*O*-sulfate^a^	3.11	233.0125	C_8_H_10_O_6_S	–1.43	99.95	PB, NPB, P
4	hydroxytyrosol 3-*O*-sulfate^a^	3.32	233.0125	C_8_H_10_O_6_S	–0.49	98.71	PB, NPB, P
5	tyrosol 4-*O*-sulfate^a^	3.35	217.0176	C_8_H_10_O_5_S	–0.85	97.84	PB, NPB, P
6	homovanillic alcohol sulfate	3.93	247.0282	C_9_H_12_O_6_S	–0.24	97.95	PB, NPB, P
7	caffeic acid 3-*O*-sulfate^a^	4.20	258.9918	C_9_H_8_O_7_S	–1.87	98.37	PB, NPB, P
8	dihydrocaffeic acid 3-*O*-sulfate^a^	4.57	261.0074	C_9_H_10_O_7_S	–1.56	99.36	P
9	*p*-coumaric acid 4-*O*-sulfate^a^	4.62	242.9969	C_9_H_8_O_6_S	0.45	97.65	PB, NPB, P
10	ferulic acid 4-*O*-sulfate^a^	4.73	273.0074	C_10_H_10_O_7_S	–2.81	97.87	PB, NPB, P
11	hydroxyphenyl propionic acid sulfate peak-1	4.80	245.0125	C_9_H_10_O_6_S	0.93	98.60	PB, NPB, P
12	dihydrocaffeic acid^a^	4.99	181.0506	C_9_H_10_O_4_	–1.84	98.29	PB, NPB, P
13	hydroxyphenyl propionic acid sulfate peak-2	5.04	245.0125	C_9_H_10_O_6_S	–2.79	94.16	PB, NPB, P
14	5-(3,4-dihydroxyphenyl)-valeric acid glucuronide	5.31	385.114	C_17_H_22_O_10_	–0.85	99.37	P
15	5-(3,4-dihydroxyphenyl valerolactone) sulfate^a^	5.44	287.0231	C_11_H_12_O_7_S	1.19	96.10	P
16	2,4-dihydroxybenzoic acid^a^	5.57	153.0193	C_7_H_6_O_4_	0.45	99.80	PB, NPB, P
17	5-(3,4-dihydroxyphenyl)-valeric acid sulfate	5.78	289.0387	C_11_H_14_O_7_S	1.23	93.85	P
18	hydroxyphenylacetic acid sulfate	5.88	230.9969	C_8_H_8_O_6_S	1.79	91.16	PB, NPB, P
19	dihydroresveratrol sulfoglucuronide	5.92	485.0759	C_20_H_22_O_12_S	0.53	94.60	P
20	3-(3′-hydroxy-4′-methoxyphenyl)propionic acid) sulfate	6.04	275.0231	C_10_H_12_O_7_S	1.32	98.40	P
21	3-(3-hydroxyphenyl) propionic acid^a^	7.34	165.0557	C_9_H_10_O_3_	–0.04	99.35	PB, NPB, P
22	resveratrol 4’-*O*-sulfate^a^	7.38	307,0282	C_14_H_12_O_6_S	0.90	96.49	P
23	ellagic acid^a^	7.53	300.9990	C_14_H_6_O_8_	–0.98	99.85	PB, NPB, P
24	dimethyl-EA glucuronide	7.60	505.0624	C_16_H_10_O_8_	1.22	99.19	P
25	5-(3-hydroxyphenyl)-valeric acid sulfate	8.03	273.0438	C_11_H_14_O_6_S	–0.89	99.01	P
26	5-(3-hydroxyphenyl)-valeric acid glucuronide	8.07	369.1191	C_17_H_22_O_9_	0.39	97.02	P
27	dihydroresveratrol 3-*O*-glucuronide^a^	8.09	405,1191	C_20_H_22_O_9_	–1.46	97.55	PB, NPB, P
28	3-(2-hydroxyphenyl) propionic acid^a^	8,48	165.0557	C_9_H_10_O_3_	–1.53	95.50	PB, NPB, P
29	resveratrol 3-*O*-sulfate^a^	8.51	307,0282	C_14_H_12_O_6_S	1.43	97.43	PB, NPB, P
30	dihydroresveratrol sulfate	8.64	309,0438	C_14_H_14_O_6_S	1.34	97.06	NPB, P
31	3-(3′-hydroxy-4′-methoxyphenyl)propionic acid)	9.05	195.0663	C_10_H_12_O_4_	–1.63	97.31	PB, NPB, P
32	hydroxyhippuric acid	9.86	194.0459	C_9_H_9_NO_4_	–2.03	92.63	P
33	phenyl propionic acid sulfate peak-1	9.88	229.0176	C_9_H_10_O_5_S	1.45	97.24	P
34	phenyl propionic acid sulfate peak-2	10.03	229.0176	C_9_H_10_O_5_S	0.07	97.33	P
35	hesperetin 7-*O*-glucuronide^a^	10.12	477.1038	C_22_H_22_O_12_	1.47	98.18	P
36	hesperetin 3′-*O*-glucuronide^a^	10.63	477.1038	C_22_H_22_O_12_	–0.78	97.87	P
37	luteolin sulfate	11.07	364.9973	C_15_H_10_O_9_S	1.43	96.84	P
38	5-(3-hydroxyphenyl)-valeric acid^a^	11.80	193.087	C_11_H_14_O_3_	0.16	93.64	PB, NPB, P
39	dimethyl ellagic acid	12.20	329.0303	C_16_H_10_O_8_	1.80	98.00	P

a Identified and quantified using their authentic standards. RT; retention time

Among the identified compounds, hydroxytyrosol and tyrosol derivatives, including hydroxytyrosol 4′-sulfate, hydroxytyrosol 3-sulfate, and tyrosol 4-sulfate, were identified in plasma, perfused brain (PB), and nonperfused brain (NPB). As shown in the Figures 2, hydroxytyrosol derivatives exhibited lower PB concentrations than NPB. Among these metabolites, tyrosol 4-sulfate followed a similar distribution pattern but had lower concentrations across all matrices (Figure 2). These results are in line with studies describing that hydroxytyrosol and tyrosol derivatives, mainly sulfates (but also glucuronides in lower proportion), were detected in plasma after olive extract consumption in rat models^53^ or after intravenous injection of hydroxytyrosol.^54^ Besides, similar to our findings, only sulfate conjugates of hydroxytyrosol and tyrosol were identified in brains collected from rats euthanized by exsanguination.^53,54^

Regarding pomegranate phenolic derivatives, although ellagic acid showed the lowest *C*_max_ in plasma (1.1 ± 0.8), it was present in the brain a 9-fold higher concentrations in NPB than PB. This agrees with a human study that reported free ellagic acid concentrations rarely exceed 100 nM in plasma, even upon a high free ellagic acid intake.^55^ Although Sprague–Dawley rats are urolithin producers (González-Sarrías et al., 2009), these microbial metabolites were absent in the plasma within the 24-h sampling period. A likely explanation could be the combination with other food extracts and/or a delay caused by capsule administration. It is known that multiple factors, including the food matrix, intestinal transit time, and the threshold of gut microbial groups involved in urolithin biosynthesis, can influence their formation.^56,57^

Other phenolic metabolites derived from hydroxycinnamic acids, likely associated with the consumption of lemon and orange extracts, such as caffeic acid 3-*O*-sulfate, *p*-coumaric acid 4-*O*-sulfate, and ferulic acid 4-*O*-sulfate, displayed distinct kinetic profiles (Figure 2). Ferulic acid 4-*O*-sulfate concentrations in plasma were approximately 3-fold higher than those of *p*-coumaric acid 4-*O*-sulfate,while the concentration of *p*-coumaric acid 4-*O*-sulfate was 14-fold higher in PB than that of ferulic acid 4-*O*-sulfate. This suggests a differential brain tissue distribution of these hydroxycinnamic acid derivatives, perhaps involving active transporters or varying affinities for BBB transport proteins. Remarkably, since the brain permeability of *p*-coumaric acid, ferulic acid, and caffeic acid have been validated via *in vitro* and *in silico* predictions,^58,59^ to the best of our knowledge, the identification of their sulfate conjugates in perfused brains after oral administration has been reported here for the first time. Besides, specific metabolites derived from citrus extracts, including hesperetin 7-*O*-glucuronide and hesperetin 3′-*O*-glucuronide, were primarily identified in plasma, with no detectable levels in PB or NPB. The absence of more polar glucuronide conjugates of hesperetin in brain tissues is consistent with previous reports conducted in *in vitro* BBB models using two different mouse (b.END5) and rat (RBE4) brain endothelial cell lines. This poor BBB permeability exhibited by the flavanone glucuronides might be attributed to their hydrophilicity and high molecular weight, thus resulting in a restricted passive diffusion across the BBB.^51,60^

Nonspecific metabolites, such as 3-(3,4-dihydroxyphenyl)propionic acid, 2,4-dihydroxybenzoic acid, 3-(3-hydroxyphenyl)propionic acid, 3-(2-hydroxyphenyl)propionic acid, and 5-(3-hydroxyphenyl)valeric acid, were consistently detected in plasma and brain at all time points. Of particular interest, 2,4-dihydroxybenzoic acid reached concentrations around 500 nM, while 5-(3-hydroxyphenyl)valeric acid was detected at approximately 5 nM in plasma. However, both metabolites showed comparable concentrations in brain tissues (Figure 2). These results are in agreement with previous studies reporting that phenolic acids can cross the BBB and influence the central nervous system.^51,58,61^

Metabolites such as 3,4-dihydroxyphenyl propionic acid sulfate and 5-(3,4-dihydroxyphenyl)valerolactone sulfate, both associated with proanthocyanidin metabolism, were exclusively detected in plasma, indicating limited BBB permeability for these compounds. This contrasts with a previous study that reported the ability to cross the BBB for 5-(3,4-dihydroxyphenyl)valerolactone sulfate *in vitro* and *in vivo*. In this sense, unlike oral administration conducted in our study, the injection of 5-(3′,4′-dihydroxyphenyl)-γ-valerolactone in animal models in that study confirmed the presence of this metabolite in the brain, which could favor its transport across the BBB.^62^ However, this approach overlooks the gastrointestinal metabolism of the phenolic compounds.

Regarding resveratrol derivatives from grape extract, resveratrol 4′-*O-*sulfate was restricted to plasma, whereas resveratrol 3-*O*-sulfate was detected in plasma, PB, and NPB. Interestingly, this latter metabolite was only present in PB at 3 and 6 h. Dihydroresveratrol 3-*O*-glucuronide reached the highest concentrations in plasma and PB (compared to all resveratrol derivatives). However, it was detectable only at specific time points (1, 3, 6, and 8 h). In this regard, few animal studies using rodent models have reported that sulfate conjugates of resveratrol are capable of crossing the BBB and reaching the perfused brain tissue at significant concentrations in the range of nmol/g of tissue.^63,64^ Notwithstanding, these studies were conducted using intravenous administration and oral doses of resveratrol, far exceeding regular dietary intake, respectively.

### Pharmacokinetic Profiles of Phenolic Metabolites in Plasma and Brain

Table 2 presents the pharmacokinetic analysis of phenolic metabolites, highlighting differences in their distribution across plasma, PB, and NPB. As expected, pharmacokinetic parameters in PB were generally different from those in NPB. For example, ferulic acid 4-*O*-sulfate and 2,4-dihydroxybenzoic acid showed significantly lower AUC values in PB compared to NPB. In addition dihydroresveratrol 3-*O*-glucuronide showed significant shorter times to reach maximum concentration (*T*_max_) in PB (3.3 ± 2.1 h) compared with NPB(6.7 ± 1.2 h)). On the contrary, caffeic acid 3-*O*-sulfate was the only metabolite to display higher *C*_max_ (1.0 ± 0.7 pmol/g tissue) and AUC (4.7 ± 1.8 pmol/g tissue·h) in PB compared to NPB, where the level reached was very low (*C*_max_: 0.24 pmol/g tissue in one animal at 3 h (Figure 2). These findings underscore that certain phenolic acids may preferentially show an enhanced transport mechanism across the BBB and reach brain regions. In this sense, previous *in vitro* and *in silico* studies support the ability of these metabolites to cross the BBB by simple diffusion influenced by the degree of lipophilicity/polarity of each metabolite. However, according to *in vivo* studies, this mechanism remains unclear,^51,60^ and alternative processes, such as the use of specific transporters as well as paracellular and vesicular transport-like mechanisms, cannot be discarded.^49,58^ Besides, the different pharmacokinetic profiles of phenolic metabolites related to their bioavailability, metabolism by gut microbiota, etc., after oral administration of extracts or foods make it difficult to identify the mechanism of transport to the brain, and these conclusions should be interpreted with caution.^58^ Altogether, it should be noted that the majority of metabolites detected in the brain were sulfate derivatives, in agreement with what has already been reported for other metabolites in animal studies, although after systemic administration of their corresponding nonconjugated compounds.^35,39,62^

**Table 2:** Pharmacokinetic Parameters of the Phenolic Metabolites Derived from Consuming the (Poly)phenol Mediterranean Diet Blend in Plasma (n = 6 per Time Point, Total n = 30) and Perfused (PB, n = 3 per Time Point, Total n = 15) and Nonperfused (NPB n = 3 per Time Point, Total n = 15) Brains^a^

	**plasma**					**PB**					**NPB**				
**metabolite**	*T*_**1/2**_**(h)**	*T*_**max**_**(h)**	*C*_**max**_**(nM)**	**AUC0-t (nM h)**	**MRT0-t (h)**	*T*_**1/2**_**(h)**	*T*_**max**_**(h)**	*C*_**max**_**(pmol/g tissue)**	**AUC0-t (pmol/g h)**	**MRT0-t (h)**	*T*_**1/2**_**(h)**	*T*_**max**_**(h)**	**Cmax (pmol/g tissue)**	**AUC0-t (pmol/g h)**	**MRT0-t (h)**
hydroxytyrosol 4’-*O*-sulfate	3.0 ± 0.6	1 ± 0	192 ± 76	793 ± 403	3.4 ± 0.5	12 ± 8.9	2.3 ± 2.5	4.8 ± 1.5	52 ± 30	18 ± 14	18 ± 14	2.3 ± 1.2	6.3 ± 2.6	84 ± 22	26 ± 22
hydroxytyrosol 3′-*O*-sulfate	4.0 ± 2.3	1.4 ± 0.9	303 ± 45	1432 ± 648	4.3 ± 1.0	7.3 ± 3.6	2.7 ± 2.9	3.4 ± 3.1	16 ± 7.8*	11 ± 5.9	7.0 ± 0.7	3.0 ± 0.0	9.7 ± 5.7	60 ± 8.4	7.1 ± 3.1
tyrosol 4’-sulfate	6.5 ± 1.8	1 ± 0	20 ± 30	122 ± 116	7.15 ± 3.3	—	2.3 ± 3.2	0.1 ± 0.08	0.9 ± 1.3	—	4.5 ± 1.1	2.0 ± 1.4	4.7 ± 6.1	31 ± 43	7.4 ± 0.4
caffeic acid 3′-*O*-sulfate	8.3 ± 4.9	8.6 ± 8.9	22 ± 21	282 ± 332	13 ± 8.8	5.2 ± 5.7	3.0 ± 0.1	1.0 ± 0.7	4.7 ± 1.8	8.7 ± 7.3		3.00	0.24	3.32	—
dihydrocaffeic 3′-*O*-sulfate	6.0 ± 1.6	4.5 ± 1.7	9.6 ± 4.5	77 ± 31	9.0 ± 2.5	ND	ND	ND	ND	ND	ND	ND	ND	ND	ND
*p*-coumaric acid 4’-*O*-sulfate	33 ± 28	3.6 ± 2.6	44 ± 47	293 ± 154	45 ± 37	23.85	19 ± 12	2.8 ± 2.4	1.1 ± 0.7	30 ± 20	—	3.0 ± 0.0	0.2 ± 0.1	1.6 ± 0.6	—
ferulic acid 4’-*O*-sulfate	12 ± 6.3	4.2 ± 1.6	129 ± 107	1114 ± 459	14 ± 5.1	—	12 ± 11	0.2 ± 0.1	3.0 ± 0.5*	—	—	1.0 ± 0.0	0.3 ± 0.2	4.8 ± 2.6	—
dihydrocaffeic acid	61 ± 94	2.6 ± 0.9	16 ± 8.6	127 ± 31	86 ± 138	—	4.5 ± 5.0	0.7 ± 0.3	11 ± 2.8*			24 ± 0.0	0.9 ± 0.1	18 ± 1	—
5-(3,4-dihydroxyphenyl valerolactone) sulfate	19 ± 29	4.5 ± 1.7	12.1 ± 5.0	113 ± 78	31 ± 44	ND	ND	ND	ND	ND	ND	ND	ND	ND	ND
2,4-dihydroxybenzoic acid	14 ± 6	12 ± 11	534 ± 211	6415 ± 1916	22 ± 11	—	10 ± 12	4.3 ± 2.8*	44 ± 17*	—	5.4 ± 3.6	14 ± 15	10 ± 1.2	92 ± 31	7.9 ± 1.1
3(3-hydroxyphenyl) propionic acid	6.9	13 ± 11	39 ± 20	481 ± 353	9.77	11 ± 6.9	3.3 ± 2.5	1.0 ± 0.5	11 ± 6.8	11 ± 6.9	20 ± 15	7.5 ± 3.1	4.0 ± 3.6	13 ± 11	11 ± 4.1
resveratrol 4’-*O*-sulfate	5.6	7.3 ± 1.2	2.9 ± 2.8	26 ± 27	10.67	ND	ND	ND	ND	ND	ND	ND	ND	ND	ND
ellagic acid	—	10 ± 12	1.1 ± 0.8	14.1 ± 7.4	103.01	—	16 ± 11	0.1 ± 0.08	1.5 ± 1.1		36.3	15 ± 13	0.9 ± 0.9	7.1 ± 7.5	56.90
dihydroresveratrol 3-*O*-glucuronide	8.1 ± 3.2	6.2 ± 2.1	93 ± 45	993 ± 558	14 ± 5.8	15 ± 18	3.3 ± 2.1*	0.4 ± 0.1	1.3 ± 0.7	23 ± 26	27 ± 1	6.7 ± 1.2	0.5 ± 0.1	5.3 ± 2.8	44 ± 1
3(2-hydroxyphenyl) propionic acid	8.26	10. ± 8.0	20 ± 11	213 ± 53	8.89	v	9.0 ± 1.4	0.6 ± 0.5	1.8 ± 2.2	—	—	3.3 ± 4.0	0.3 ± 0.04	1.1 ± 0.8	—
resveratrol 3′-*O*-sulfate	3.9 ± 4.3	5.2 ± 2.2	2.2 ± 2.6	8.4 ± 10	8.5 ± 5.5	—	5.0 ± 1.7	0.1 ± 0.01	0.1 ± 0.1			15 ± 13	0.2 ± 0.04	2.2 ± 0.1	—
hesperetin 7’-*O*-glucuronide	7.4 ± 3.4	4.2 ± 2.8	4.0 ± 0.7	36 ± 12	11 ± 5.8	ND	ND	ND	ND	ND	ND	ND	ND	ND	ND
hesperetin 3′-*O*-glucuronide	14.80	5.7 ± 2.7	2.7 ± 0.9	11 ± 1.3	23.48	ND	ND	ND	ND	ND	ND	ND	ND	ND	ND
5-(3-hydroxyphenyl)-valeric	29 ± 32	7.4 ± 9.5	4.8 ± 1.9	55 ± 11	43 ± 45	39 ± 19	2.0 ± 1.2	2.4 ± 0.8	30 ± 6.5	323 ± 477	70 ± 46	2.3 ± 1.2	3.4 ± 0.7	33 ± 4.8	102 ± 62

a ND, not detected; —, not determined due to insufficient data points; *T*_1/2_, the time required for the concentration to decrease to half of its original value; *T*_max_, time at which maximum concentration occurs; *C*_max_, maximum concentration observed; AUC0–t, the area under the concentration–time curve from dosing to the final quantifiable concentration; MRT0–t, mean residence time from dosing to the final quantifiable concentration. Asterisks indicate significant differences between parameters in PB and NPB (*P* < 0.05).

In plasma, 2,4-dihydroxybenzoic acid reached the highest concentration among all compounds (*C*_max_: 534 ± 211 nM; AUC: 6415 ± 1916 nM·h), followed by hydroxytyrosol 3-*O*-sulfate (*C*_max_: 303 ± 45 nM; AUC: 1432 ± 648 nM·h). Remarkably, hydroxytyrosol 4-*O*-sulfate showed lower *C*_max_ values in plasma (192 ± 76 nM) than its isomer, hydroxytyrosol 3-*O*-sulfate. However, hydroxytyrosol 4-*O*-sulfate exhibited higher levels in PB than hydroxytyrosol-3*-O*-sulfate, indicating distinct distribution patterns toward the brain. In agreement with our results, previous studies have reported the rapid absorption and brain distribution of sulfate conjugates of hydroxytyrosol and tyrosol, reaching a *T*_max_ around 2 h after oral ingestion.^53^ Additionally, other metabolites showed notable differences, such as ferulic acid 4-*O*-sulfate and *p*-coumaric acid 4-*O*-sulfate. Ferulic acid 4-*O*-sulfate had a plasma *C*_max_ of 129 nM in plasma, and *p*-coumaric acid 4-*O*-sulfate 44 nM. However, *p*-coumaric acid 4-*O*-sulfate showed a 14-fold higher concentration in PB than ferulic acid 4-*O*-sulfate. Furthermore, the pharmacokinetics of other metabolites, such as tyrosol sulfate and 5-(3-hydroxyphenyl)-valeric revealed comparable patterns. Thus, tyrosol sulfate was detected at higher levels in plasma compared to 5-(3-hydroxyphenyl)-valeric. However, the latter exhibited a much higher concentration in PB than tyrosol sulfate. Finally, 5-(3,4-dihydroxyphenyl valerolactone) sulfate displayed long half-life in plasma (*t*_1/2_ = 20 ± 29 h) and mean residence time (MRT = 31 ± 44), indicative of its potential for prolonged systemic effects.^65^ However, its distribution in brain tissues was undetectable in both PB and NPB, emphasizing its limited BBB permeability.

### Phenolic Metabolites Can Be Transported across the BBB Endothelium

**Figure 3:**
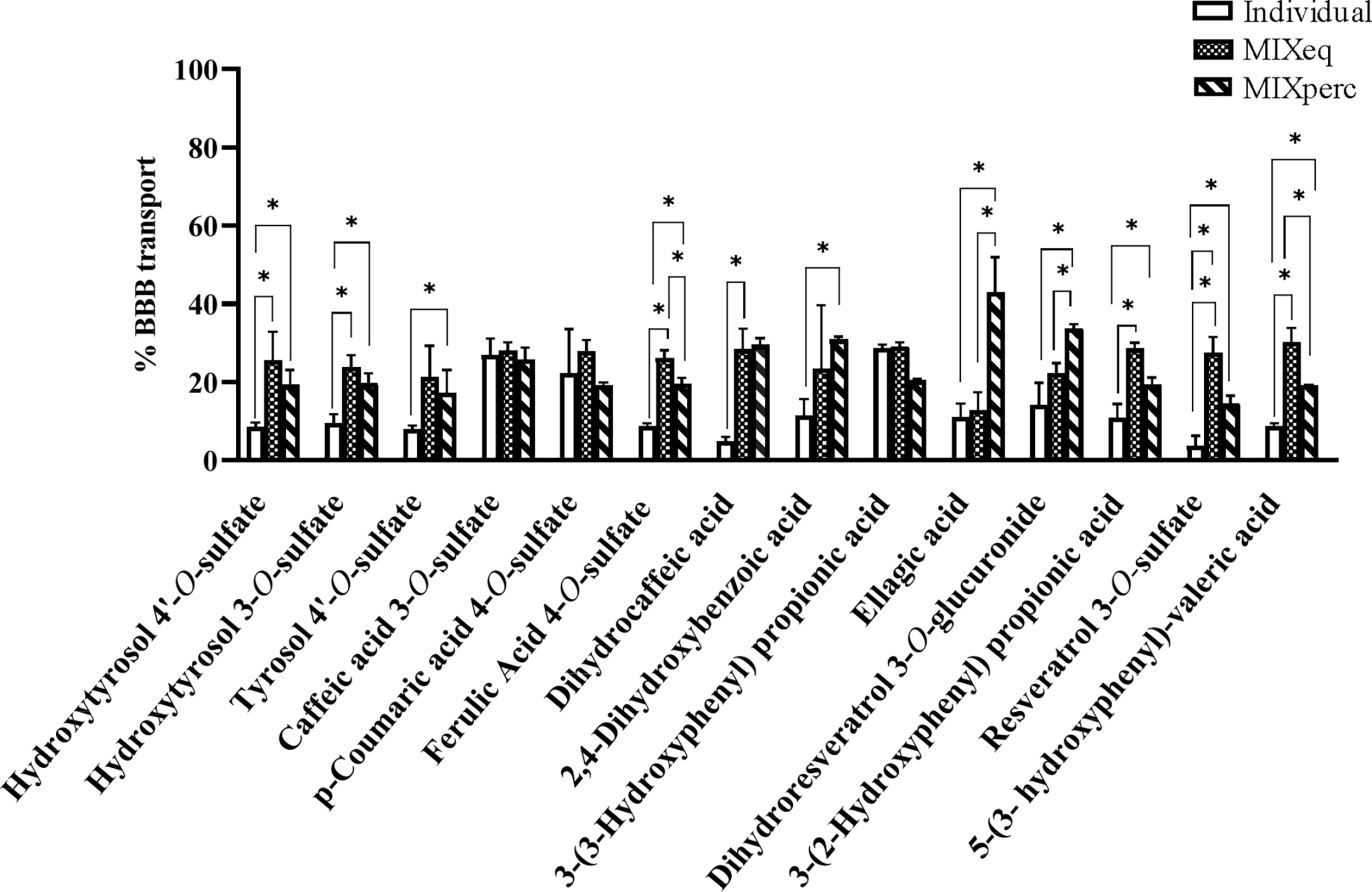
Transport efficiency of phenolic metabolites across the BBB. Human brain microvascular endothelial cells (HBMEC) were seeded onto semipermeable membranes. At confluence, cells were treated, for 2 h, with 2.5 μM individual phenolic compounds or two different mixtures: (i) a representative mixture of all compounds detected in PB with the same concentration of each one (MIXeq), and (ii) a representative mixture reflecting the percentage of the real concentrations (Cmax) observed for each compound in PB (MIXperc). Detailed composition of both mixtures is given in the Supporting Information, Table S3. Media from the upper and lower compartments were collected, and endothelial transport was evaluated by UPLC-qTOF-MS. The transport percentage (%) was determined by the concentration ratio in the lower compartment to the total concentration (upper and lower compartments). All values are presented as mean ± SD from three independent experiments.

The transport efficiency of phenolic metabolites across the BBB was evaluated using a well-established *in vitro* model of human brain microvascular endothelial cells (HBMEC).^66^ As shown in Figure 3, the transport percentage of individual phenolic compounds and two different mixtures, equal concentration and physiological concentration reflecting the percentage of the actual concentrations (*C*_max_) observed for each compound in PB (Supporting Information, Table S3), revealed significant differences, supporting the hypothesis that specific structural properties influence BBB permeability.

Among the individual compounds, caffeic acid 3-*O*-sulfate, *p*-coumaric acid 4-*O*-sulfate and 3-(3-hydroxyphenyl) propionic acid exhibited the highest transport efficiencies, with percentages exceeding 20%, indicating their ability to cross the BBB readily. In contrast, dihydrocaffeic acid and resveratrol 3-*O*-sulfate showed the lowest transport percentages (<5%), reflecting their limited permeability across the endothelial layer. These results are consistent with the *in vivo* observations, as both compounds exhibited substantial concentrations in plasma but low levels in the brain (Table 2). Besides, these findings are also in line with previous studies indicating that metabolites such as tyrosol sulfate or resveratrol sulfate can cross this *in vitro* BBB model.^38,67^ Additionally, the transport across the BBB of other metabolites, such as phenolic acids, has been observed in different *in vitro* BBB models.^51,68^

When comparing the two mixtures, the physiological concentration mixture (MIXperc) demonstrated significantly higher transport efficiencies for compounds such as ellagic acid, dihydroresveratrol 3-*O*-glucuronide. In contrast, resveratrol 3-*O*-sulfate and 5-(3-hydroxyphenyl)-valeric acid showed lower transport percentages. Interestingly, each compound exhibited a distinct transport behavior depending on the mixture, while most metabolites did not show significant differences between the two conditions. These results highlight the potential role of relative concentrations in modulating BBB permeability for specific phenolic metabolites. Interestingly, some metabolites, such as caffeic acid 3-*O*-sulfate, *p*-coumaric acid 4-*O*-sulfate and 3-(3-hydroxyphenyl)propionic acid, displayed relatively consistent transport efficiencies when tested in both mixtures and as individual molecules, suggesting less influence of the concentration on their BBB permeability. These results underscore the differential behavior of phenolic metabolites in crossing the BBB, with specific compounds and mixture compositions showing enhanced transport capacities.

Overall, the present study, combining *in vivo* and *in vitro* models, provides compelling evidence of how circulating phenolic metabolites derived from a Mediterranean diet rich in (poly)phenols can effectively cross the BBB and reach brain tissues. These findings set the basis for understanding the neuroprotective potential of circulating phenolic metabolites from pomegranate, lemon, orange, grape, and olive, reinforcing their role in preventing or delaying the onset of neurodegenerative disorders. Hence, the fact that circulating phenolic metabolites reach brain tissues mostly in their metabolized forms warrants future research focused on identifying the most neuroprotective metabolites and optimizing their bioavailability through diet or supplementation.^23,59^ Although in our study the use of capsules enabled accurate dosing and standardized formulation of the (poly)phenol-rich extract, it is important to note that the absence of a complex food matrix may influence polyphenol absorption and metabolism. Therefore, matrix effects should be considered when translating these findings to real dietary conditions.

Nonetheless, even if circulating phenolic metabolites cannot cross the BBB and reach the brain in considerable concentrations, they remain bioavailable in the bloodstream and may still exert beneficial effects at systemic levels.^69,70^ While this study focuses on the direct brain bioavailability of phenolic metabolites, it is worth noting that central effects may also be mediated indirectly through gut–brain axis modulation. Polyphenols can influence brain aging not only by crossing the BBB, but also by reshaping gut microbiota composition, enhancing barrier integrity, and generating microbial-derived metabolites that affect the CNS via immune, endocrine, or neural pathways.^71^ Finally, these findings also emphasize the complexity of phenolic metabolite transport across the BBB, highlighting that the characteristics of individual compounds and the composition of metabolites influence brain accessibility. Therefore, further research is needed to investigate the mechanisms underlying BBB transport to optimize the neuroprotective potential of dietary polyphenols.

## Supporting Information Available

**Figure S1**. Phenolic metabolite profiles in plasma compared to the brain, but not quantified in plasma (black), perfused brain (PB, blue), and nonperfused brain (NPB, gray) derived from the consumption of a (poly)phenol Mediterranean blend. Values are expressed as area values with standard deviations (SD). Data correspond to plasma samples (*n* = 6) and brain samples (*n* = 3) for each group of animals. **Table S1.** Main phenolic compounds administered to each animal by gavage. **Table S2.** Main phenolic compounds and derived metabolites searched **Table S3.** Mixtures of all phenolic compounds detected in the perfused brain using equal concentrations (MIXeq) or percentages according to the Cmax values of each one (MIXperc). BBB transport of each one alone or using these mixtures was assayed in HBMEC cells, as shown in Figure 3 (PDF)

## Abbreviations Used

ACN: acetonitrile
AUC: the total area under the curve
AUC0-t: AUC from the initial time point (0 h) to the final time point (24 h)
AUC0-∞: AUC from the initial time point (0 h) to infinite time
BBB: blood-brain barrier
*C*_max_: maximum peak concentration
EIC: extracted ion chromatogram
ESI: electrospray ionization
FBS: fetal bovine serum
HBMECs: Human brain microvascular endothelial cells
HBSS: Hanks’ balanced salt solution
HED: human equivalent dose
MeOH: methanol
MRT: mean residence time
MS: mass spectrometry
ND: neurodegenerative diseases
NPB: nonperfused brain
PB: perfused brain
PBS: phosphate buffer saline
SD: standard deviation
*T*_1/2_: half-life
TEER: transepithelial electrical resistance
*T*_max_: time to reach *C*_max_
UPLC: ultrahigh-performance liquid chromatography
