# Supplementary material for: Metabolic Profiling of a Mediterranean-Inspired (Poly)phenol-Rich Mixture in the Brain: Perfusion Effect and In Vitro Blood–Brain Barrier Transport Validation
Source: J Agric Food Chem. 2025 Apr 24;73(18):11056–66. doi: 10.1021/acs.jafc.5c02288 (PMC12063173; doi:10.1021/acs.jafc.5c02288)
Supplement: Supplementary file 1 — jf5c02288_si_001.pdf [file jf5c02288_si_001.pdf]

Supporting Information

Metabolic Profiling of a Mediterranean-Inspired (Poly)phenol-Rich Mixture in the Brain: Perfusion Effect and *In Vitro* Blood-Brain Barrier Transport Validation

María Ángeles Ávila-Gálvez[1]*, Beatriz Garay-Mayol[1], Alicia Marín[1], María Alexandra Brito[2], Juan Antonio Giménez-Bastida[1], Juan Carlos Espín[1] and Antonio González-Sarrías[1]*

[1] Laboratory of Food & Health, Research Group on Quality, Safety and Bioactivity of Plant Foods, CEBAS-CSIC, 30100 Campus de Espinardo, Murcia, Spain

[2] Research Institute for Medicines (iMed.ULisboa), Faculty of Pharmacy, Universidade de Lisboa, Av. Prof. Gama Pinto, 1649-003 Lisbon, Portugal; Department of Pharmaceutical Sciences and Medicines, Faculty of Pharmacy, Universidade de Lisboa, Av. Prof. Gama Pinto, 1649-003 Lisbon, Portugal.

*Corresponding authors: M.A. Ávila-Gálvez, mavila@cebas.csic.es; A. González-Sarrías, agsarrias@cebas.csic.es

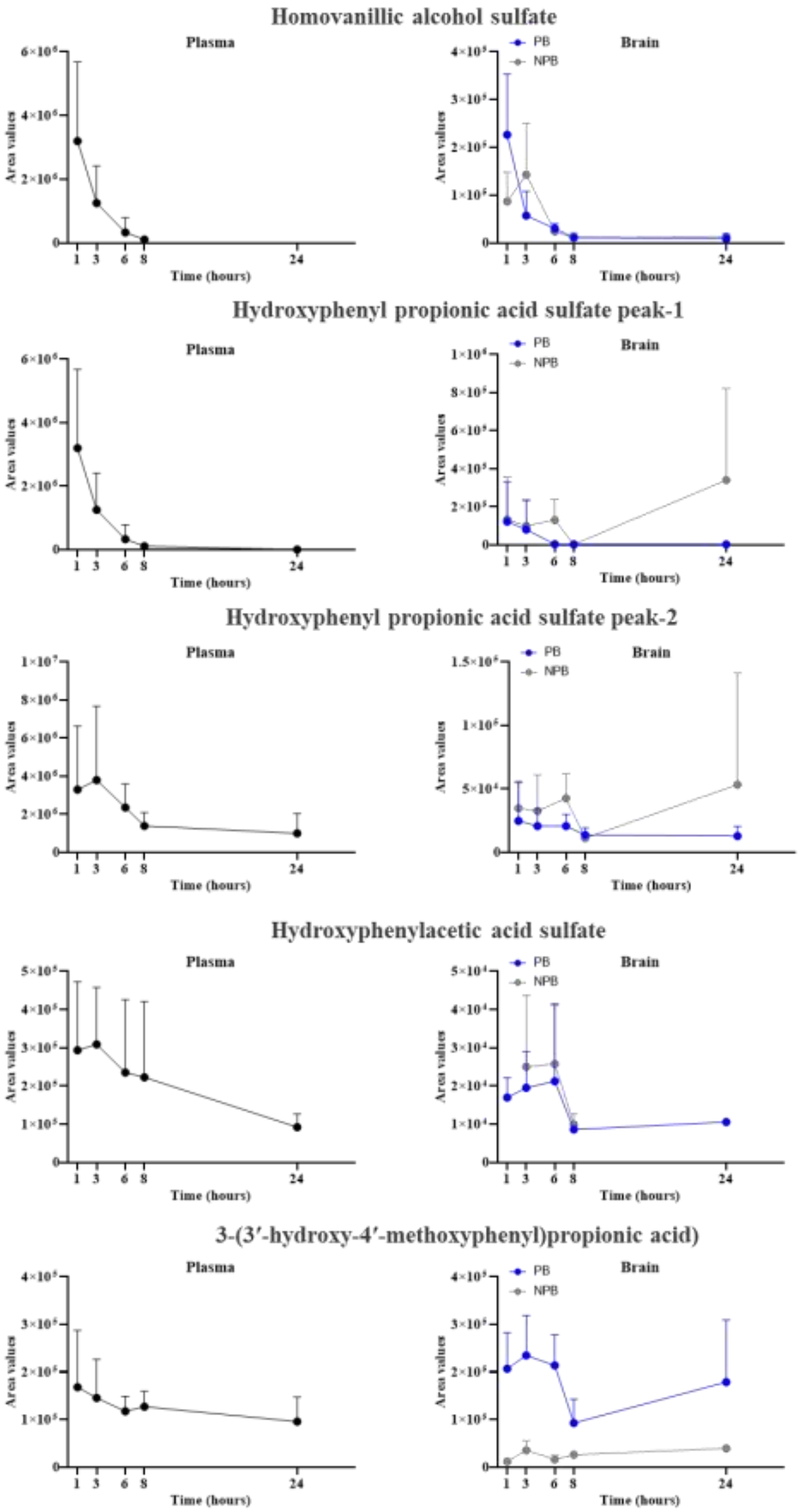

Homovanillic alcohol sulfate
Plasma
Brain
PB
NPB
Area values
Time (hours)
Hydroxyphenyl propionic acid sulfate peak-1
Plasma
Brain
PB
NPB
Area values
Time (hours)
Hydroxyphenyl propionic acid sulfate peak-2
Plasma
Brain
PB
NPB
Area values
Time (hours)
Hydroxyphenylacetic acid sulfate
Plasma
Brain
PB
NPB
Area values
Time (hours)
3-(3′-hydroxy-4′-methoxyphenyl)propionic acid
Plasma
Brain
PB
NPB
Area values
Time (hours)

Figure S1. Phenolic metabolite profiles in plasma compared to the brain, but not quantified in plasma (black), perfused brain (PB, blue), and nonperfused brain (NPB, grey) derived from the consumption of a (poly)phenol Mediterranean blend. Values are expressed as area values with standard deviations (SD). Data correspond to plasma samples (n=6) and brain samples (n=3) for each group of animals.

Supplementary Tables

Table S1. Main phenolic compounds administered to each animal by gavage.

Compound	Class	RT	*m/z*⁻	MS/MS	λ_{max}	mg/g extract	mg/g blend	mg/capsule
**Lemon**								
Luteolin-6,8-di-C-glucoside[a]	Flavone	19.61	609	519/489/369	270/336	53.90±3.84	4.56±0.32	0.12±0.01
Apigenin 6,8-di-C-glucoside[a]	Flavone	20.81	593	503/473/353	270/332	48.07±5.00	4.07±0.42	0.11±0.01
Eriocitrin	Flavanone	22.70	595	459/329/287	284/334	114.53±8.10	9.69±0.69	0.25±0.02
Limocitrin-neohesperidoside[b]	Flavonol	25.29	653	345/330/301	274/352	9.08±0.64	0.77±0.05	0.02±0.001
Hesperidin	Flavanone	26.65	609	301	280/340	63.56±6.17	5.38±0.52	0.14±0.01
**Orange**								
Hesperidin	Flavanone	25.83	609	301	280/340	657.23±24.27	55.61±2.05	1.45±0.05
**Pomegranate (rich in ellagic acid)**								
Ellagic acid	Ellagic acid	23.02	301	256/184	254/360	599.63±43.94	123.62±9.06	3.21±0.24
**Pomegranate (rich in punicalagin)**								
Punicalagin	Ellagitannin	11.55/13.23	1083	781/721/601	258/372	144.76±15.71	7.46±0.81	0.19±0.02
Ellagic acid	Ellagic acid	22.94	301	256/184	254/360	67.01±6.86	3.45±0.35	0.09±0.009
**Olive**								
Hydroxytyrosol	Benzene diols	14.34	153	123	274	120.71±5.93	31.11±1.53	0.81±0.04
**Grape**								
trans-Resveratrol	Stilbene	28.91	227	185	306	1,097±36.23	50.65±1.67	1.32±0.04
Catechin adduct	Procyanidin	4.40	413	287/125	280	149.93±7.44	12.69±0.63	0.33±0.02

Catechin monomer	Procyanidin	6.61	289	271/179	280	61.46±3.33	5.20±0.28	0.14±0.01
Catechin gallate adduct	Procyanidin	8.30	565	439/261	280	19.00±0.58	1.61±0.05	0.04±0.001
Epicatechin monomer	Procyanidin	9.82	289	271/179	280	43.81±3.29	3.71±0.28	0.10±0.01
Epicatechin gallate adduct	Procyanidin	11.30	565	439/261	280	3.68±1.36	0.31±0.12	0.01±0.003
Catechin gallate	Procyanidin	15.22	441	397/169	280	9.40±1.19	0.80±0.10	0.02±0.003
Epicatechin gallate monomer	Procyanidin	16.11	441	397/169	280	0.98±0.13	0.08±0.01	0.002±0.0003
					**Total phenolics**	**3,264±174.01**	**320.77±18.94**	**8.35±0.50**

Extraction and HPLC methodology followed a protocol described elsewhere (Ávila-Gálvez et al., 2019). The compounds were identified using the elution order, UV spectra, molecular weight, MS/MS fragmentation, and chromatographic comparison with authentic standards. Values are shown as mean ± SD (n=3). RT, retention time. * The composition of the procyanidins in the grape extract was determined by phloroglucinolysis. [a]Quantified at 340 nm with apigenin. [b] Quantified at 360 nm with rutin.

Table S2. Main phenolic compounds and derived metabolites searched

**Phenolic metabolites derived from orange/lemon extracts**							
Nº	Compound	**Formula**	**Exact mass (*m/z*⁻)**	Nº	Compound	**Formula**	**Exact mass (*m/z*⁻)**
1	Naringenin	C15H12O5	271.0612	30	Hydroxyphenyl acetic acid sulfate	C8H8O6S	230.9969
2	Naringenin glucuronide	C21H20O11	447.0933	31	Phenyl acetic acid	C8H8O2	135.0452
3	Naringenin diglucuronide	C27H28O17	623.1254	32	Phenyl acetic acid glucuronide	C14H16O8	311.0772
4	Naringenin sulfoglucuronide	C21H20O14S	527.0501	33	Phenyl acetic acid sulfate	C8H8O5S	215.0020
5	Naringenin sulfate	C15H12O8S	351.0180	34	Dihydroxybenzoic acid	C7H6O4	153.0193
6	Hesperetin	C16H14O6	301.0718	35	Dihydroxybenzoic acid glucuronide	C13H14O10	329.0514
7	Hesperetin glucuronide	C22H22O12	477.1038	36	Dihydroxybenzoic acid sulfate	C7H6O7S	232.9761
8	Hesperetin diglucuronide	C28H30O18	653.1359	37	Hydroxybenzoic acid	C7H6O3	137.0244
9	Hesperetin sulfoglucuronide	C22H22O15S	557.0607	38	Hydroxybenzoic acid glucuronide	C13H14O9	313.0565
10	Hesperetin sulfate	C16H14O9S	381.0286	39	Hydroxybenzoic acid sulfate	C7H6O6S	216.9812
11	Eriodictyol	C15H12O6	287.0561	40	Benzoic acid	C7H6O2	121.0295
12	Eriodictyol glucuronide	C21H20O12	463.0882	41	Benzoic acid glucuronide	C13H14O8	297.0616
13	Eriodictyol diglucuronide	C27H28O18	639.1203	42	Benzoic acid sulfate	C7H6O5S	200.9683
14	Eriodictyol sulfoglucuronide	C21H20O15S	543.0450	43	*p*-Coumaric acid	C9H8O3	163.0401
15	Eriodictyol sulfate	C15H12O9S	367.0129	44	*p*-Coumaric acid glucuronide	C15H16O9	339.0722
16	Dihydroxyphenyl propionic acid	C9H10O4	181.0506	45	p-Coumaric acid sulfate	C9H8O6S	242.9969
17	Dihydroxyphenyl propionic acid glucuronide	C15H18O10	357.0827	46	Caffeic acid	C9H8O4	179.0350
18	Dihydroxyphenyl propionic acid sulfate	C9H10O7S	261.0074	47	Caffeic acid glucuronide	C15H16O10	355.0671

19	Hydroxyphenyl propionic acid	C9H10O3	165.0557	48	Caffeic acid sulfate	C9H8O7S	258.9918
20	Hydroxyphenyl propionic acid glucuronide	C15H18O9	341.0878	49	Ferulic acid	C10H10O4	193.0506
21	Hydroxyphenyl propionic acid sulfate	C9H10O6S	245.0125	50	Ferulic acid glucuronide	C16H18O10	369.0827
22	Phenyl propionic acid	C9H10O2	149.0608	51	Ferulic acid sulfate	C10H10O7S	273.0074
23	Phenyl propionic acid glucuronide	C15H18O8	325.0929	52	Dihydroisoferulic acid	C10H12O4	195.0663
24	Phenyl propionic acid sulfate	C9H10O5S	229.0176	53	Dihydroisoferulic acid glucuronide	C16H20O10	371.0984
25	Dihydroxyphenyl acetic acid	C8H8O4	167.0350	54	Dihydroisoferulic acid sulfate	C10H12O7S	275.0231
26	Dihydroxyphenyl acetic acid glucuronide	C14H16O10	343.0671	55	Hydroxyhippuric acid	C9H9NO4	194.0459
27	Dihydroxyphenyl acetic acid sulfate	C8H8O7S	246.9918	56	Hydroxyhippuric acid glucuronide	C15H17NO10	370.0780
28	Hydroxyphenyl acetic acid	C8H8O3	151.0401	57	Hydroxyhippuric acid sulfate	C9H9NO7S	274.0027
29	Hydroxyphenyl acetic acid glucuronide	C14H16O9	327.0722				
**Phenolic metabolites derived from pomegranate extract**							
58	Urolithin-A	C13H8O4	227.035	74	Ellagic acid (EA)	C14H6O8	300.999
59	Urolithin-A glucuronide	C19H16O10	403.0671	75	Ellagic acid glucuronide	C20H14O14	477.0311
60	Urolithin-A sulfate	C13H8O7S	306.9918	76	Ellagic acid sulfate	C14H6O11S	380.9558
61	Urolithin-A sulfoglucuronide	C19H16O13S	483.0239	77	Methyl-EA	C15H8O8	315.0146
62	Urolithin-B	C13H8O3	211.0401	78	Methyl-EA glucuronide	C21H16O14	491.0467
63	Urolithin-B glucuronide	C19H16O9	387.0722	79	Methyl-EA sulfate	C15H8O11S	394.9715
64	Urolithin B sulfate	C13H8O6S	290.9969	80	Dimethyl-EA	C16H10O8	329.0303
65	Urolithin-C	C13H8O5	243.0299	81	Dimethyl-EA glucuronide	C16H10O8	505.0624

66	Urolithin-C glucuronide	C19H16O11	419.062	82	Dimethyl-EA sulfate	C16H10O11S	408.9871
67	Urolithin-C sulfate	C13H8O8S	322.9867	83	Gallic acid	C7H6O5	169.0142
68	Urolithin-D	C13H8O6	259.0248	84	Valoneic acid dilactone	C21H10O13	469.0049
69	Urolithin-D glucuronide	C19H16O12	435.0569	85	Gallagic acid dilactone	C28H10O16	600.9896
70	Urolithin-D sulfate	C13H8O9S	338.9816	86	Methyl-Urolithin-C	C14H11O5	258.0534
71	Urolithin-M5	C13H8O7	275.0197	87	Methyl-Urolithin-C glucuronide	C20H19O11	434.0855
72	Urolithin-M5 glucuronide	C19H16O13	451.0518	88	Methyl-Urolithin-C sulfate	C14H11O8S	338.0102
73	Urolithin-M5 sulfate	C13H8O10S	354.9765				
**Phenolic metabolites derived from olive extract**							
89	Hydroxytyrosol	C8H10O3	153.0557	101	Oleuropein	C19H22O8	377.1242
90	Hydroxytyrosol glucuronide	C14H18O9	329.0878	102	Oleuropein glucuronide	C25H30O14	553.1563
91	Hydroxytyrosol sulfate	C8H10O6S	233.0125	103	Oleuropein sulfate	C19H22O11S	457.081
92	Hydroxytyrosol sulfoglucuronide	C14H18O12S	409.0446	104	Ligstroside	C19H22O7	361.1293
93	Homovanillic alcohol	C9H12O3	167.0714	105	Ligstroside glucuronide	C25H30O13	537.1614
94	Homovanillic alcohol glucuronide	C15H20O9	343.1035	106	Ligstroside sulfate	C19H22O10S	441.0861
95	Homovanillic alcohol sulfate	C9H12O6S	247.0282	107	Elenolic acid	C11H14O6	241.0718
96	Hydroxytyrosol-acetate	C10H12O4	195.0663	108	Elenolic acid glucuronide	C17H22O12	417.1038
97	Hydroxytyrosol-acetate glucuronide	C16H20O10	371.0984	109	Elenolic acid sulfate	C11H14O9S	321.0286
98	Hydroxytyrosol-acetate sulfate	C10H12O7S	275.0231	110	Luteolin	C15H10O6	285.0405
98	Tyrosol	C8H10O2	137.0608	111	Luteolin glucuronide	C21H18O12	461.0725
99	Tyrosol glucuronide	C14H18O8	313.0929	112	Luteolin sulfate	C15H10O9S	364.9973
100	Tyrosol sulfate	C8H10O5S	217.0176				
**Phenolic metabolites derived from grape**							

113	*t*-Resveratrol	C14H12O3	227.0714	138	Epicatechin dimethyl ether sulfate	C17H18O9S	397.0599
114	*t*-Resveratrol glucuronide	C20H20O9	403.1035	139	3,4-diHPP-2-ol	C15H16O6	291.0874
115	*t*-Resveratrol sulfate	C14H12O6S	307.0282	140	3,4-diHPP-2-ol glucuronide	C21H24O12	467.1195
116	*t*-Resveratrol disulfate	C14H12O9S2	386.985	141	3,4-diHPP-2-ol sulfate	C15H16O9S	371.0442
117	*t*-Resveratrol diglucuronide	C26H28O15	579.1355	142	3-HPP-2-ol	C15H16O5	275.0925
118	*t*-Resveratrol sulfoglucuronide	C20H20O12S	483.0603	143	3-HPP-2-ol glucuronide	C21H24O11	451.1246
119	Dihydro-resveratrol	C14H14O3	229.087	144	3-HPP-2-ol sulfate	C15H16O8S	355.0493
120	Dihydro-resveratrol glucuronide	C20H22O9	405.1191	145	5-(3,4-dihydroxyphenyl valerolactone)	C11H12O4	207.0663
121	Dihydro-resveratrol sulfate	C14H14O6S	309.0438	146	5-(3,4-dihydroxyphenyl valerolactone) glucuronide	C17H20O10	383.0984
122	Dihydro-reveratrol sulfoglucuronide	C20H22O12S	485.0759	147	5-(3,4-dihydroxyphenyl valerolactone) sulfate	C11H12O7S	287.0231
123	Lunularin	C14H14O2	213.0921	148	5-(3-Hydroxyphenyl)-γ-valerolatone	C11H12O3	191.0714
124	Lunularin glucuronide	C20H24O8	391.1398	149	5-(3-Hydroxyphenyl)-γ-valerolatone glucuronide	C17H20O9	367.1035
125	Lunularin sulfate	C14H14O5S	293.0489	150	5-(3-Hydroxyphenyl)-γ-valerolatone sulfate	C11H12O6S	271.0282
126	3,4-Dihydroxy-trans-stilbene	C14H12O2	211.0765	151	5-Phenyl-γ-valerolatone	C11H12O2	175.0765
127	3,4-Dihydroxy-trans-stilbene glucuronide	C20H20O8	387.1085	152	5-Phenyl-γ-valerolatone glucuronide	C17H20O8	351.1085
128	3,4-Dihydroxy-trans-stilbene sulfate	C14H12O5S	291.0333	153	5-Phenyl-γ-valerolatone sulfate	C11H12O5S	255.0333
129	Catechin or (Epi)catechin	C15H14O6	289.0718	154	5-(3,4-Dihydroxyphenyl)-valeric acid	C11H14O4	209.0819
130	Catechin or (Epi)catechin glucuronide	C21H22O12	465.1038	155	5-(3,4-Dihydroxyphenyl)-valeric acid glucuronide	C17H22O10	385.114
131	Epicatechin sulfate	C15H14O9S	369.0286	156	5-(3,4-Dihydroxyphenyl)-valeric	C11H14O7S	289.0387

					acid sulfate		
132	Epicatechin glucu sulfate	C21H22O15S	545.0607	157	5-(3-hydroxyphenyl)-valeric acid	C11H14O3	193.087
133	Epicatechin methyl ether	C16H16O6	303.0874	158	5-(3-hydroxyphenyl)-valeric acid glucuronide	C17H22O9	369.1191
134	Epicatechin methyl ether glucuronide	C22H24O12	479.1195	159	5-(3-hydroxyphenyl)-valeric acid sulfate	C11H14O6S	273.0438
135	Epicatechin methyl ether sulfate	C16H16O9S	383.0442	160	5-phenyl-valeric acid	C11H14O2	177.0921
136	Epicatechin dimethyl ether	C17H18O6	317.1031	161	5-phenyl-valeric acid glucuronide	C17H22O8	353.1242
137	Epicatechin dimethyl ether glucuronide	C23H26O12	493.1351	162	5-phenyl-valeric acid sulfate	C11H14O5S	257.0489

Table S3. Mixtures of all phenolic compounds detected in the perfused brain using equal concentrations (MIXeq) or percentages according to the Cmax values of each one (MIXperc). BBB transport of each one alone or using these mixtures was assayed in HBMEC cells, as shown in **Figure 3**.

**Compound**	**MIXeq**	**MIXperc**
Hydroxytyrosol 4´-*O*-sulfate	7.14%	21.91%
Hydroxytyrosol 3-*O*-sulfate	7.14%	15.52%
Tyrosol 4-*O*-sulfate	7.14%	0.46%
Caffeic acid 3-*O*-sulfate	7.14%	4.56%
p-Coumaric acid 4-*O*-sulfate	7.14%	12.78%
Ferulic Acid 4-*O*-sulfate	7.14%	0.91%
Dihydrocaffeic acid	7.14%	3.19%
2,4-Dihydroxybenzoic acid	7.14%	19.63%
3-(3-Hydroxyphenyl) propionic acid	7.14%	4.56%
Ellagic acid	7.14%	0.50%
Dihydroresveratrol 3-*O*-glucuronide	7.14%	1.83%
3-(2-Hydroxyphenyl) propionic acid	7.14%	2.74%
t-Resveratrol 3-*O*-sulfate	7.14%	0.46%
5-(3-Hydroxyphenyl)-valeric acid	7.14%	10.95%
**Final concentrations assayed**	**2.5 µM**	**2.5 µM**

References

Ávila-Gálvez, M. Á.; García-Villalba, R.; Martínez-Díaz, F.; Ocaña-Castillo, B.; Monedero-Saiz, T.; Torrecillas-Sánchez, A.; Abellán, B.; González-Sarrías, A.; Espín, J. C. Metabolic Profiling of Dietary Polyphenols and Methylxanthines in Normal and Malignant Mammary Tissues from Breast Cancer Patients. *Mol. Nutr. Food Res*. **2019**, *63*, e1801239.
